# The modifying effect of chronological age on the predictive value of vascular aging indicators for the long-term cardiovascular events risk

**DOI:** 10.1038/s41440-025-02503-6

**Published:** 2026-01-15

**Authors:** Tianhui Dong, Fangfang Fan, Jia Jia, Hongyu Chen, Zhichen Dong, Qiwen Zheng, Jianping Li, Yong Huo, Yan Zhang

**Affiliations:** 1 Department of Cardiology, Peking University First Hospital, Beijing, China; 2 Institute of Cardiovascular Disease, Peking University First Hospital, Beijing, China; 3 Hypertension Precision Diagnosis and Treatment Research Center, Peking University First Hospital, Beijing, China; 4 National Genomics Data Center, China National Center for Bioinformation, Beijing, China; 5 Beijing Institute of Genomics, Chinese Academy of Sciences, Beijing, China; 6 State Key Laboratory of Vascular Homeostasis and Remodeling, Peking University, Beijing, China; 7 NHC Key Laboratory of Cardiovascular Molecular Biology and Regulatory Peptides, Peking University, Beijing, China

**Keywords:** Brachial-ankle pulse wave velocity, Cardiovascular events, Chronological age, Vascular aging

## Abstract

Whether chronological age affects the ability of vascular aging indicators to predict cardiovascular events risk remains unknown. This study sought to examine whether the predictability of vascular aging indicators is better in middle-aged participants than older participants. This prospective cohort study included 8163 participants from a community-based atherosclerosis cohort in Beijing, China. Vascular age (VA) was defined as the predicted age in a multivariable regression model including cardiovascular risk factors and pulse wave velocity. Residuals by regressing VA on chronological age were defined as ∆-age, reflecting vascular aging. We used Cox proportional hazard regression model to examine the association between ∆-age and the risk of cardiovascular events in different chronological age groups. Of all participants, 5691 (69.7%) were between 40 and 60 years old, and 2472 (30.3%) were over 60 years old. During a median 9.9-year follow-up period, 818 (10%) endpoints were observed. After adjusting for confounders, ∆-age was positively associated with the risk of cardiovascular events in middle-aged participants (HR: 1.13, 95% CI: 1.07–1.21; p < 0.001), whereas no significant association was observed in older participants (HR: 1.03, 95% CI: 0.99–1.06; p = 0.148). Interaction analysis in total participants showed that chronological age significantly modified the relationship between ∆-age and the risk of cardiovascular events (p = 0.017). Our findings indicate that the predictive ability of residuals between VA and chronological age for the risk of cardiovascular events is better in middle-aged people than that in older people. The VA assessment may be more valuable to the middle-aged population.

**Figure:**
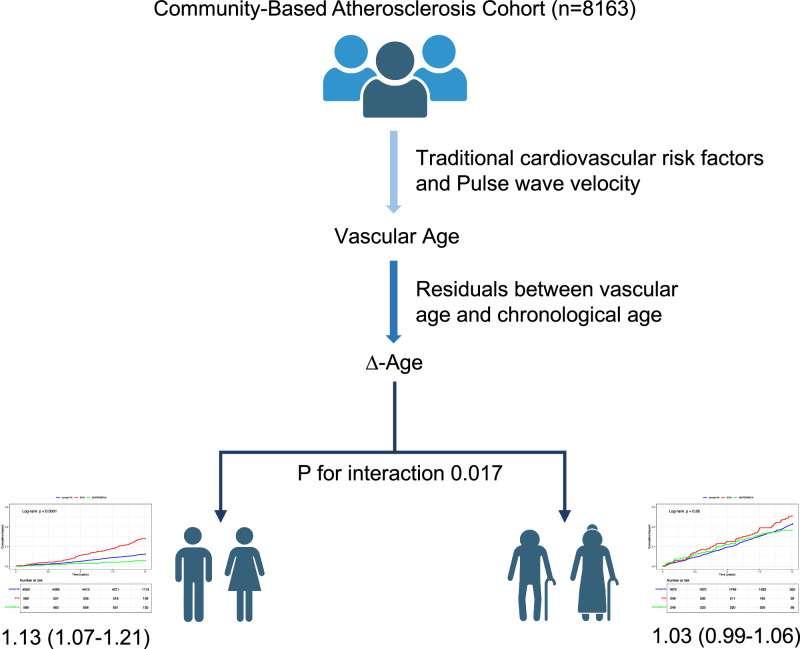
The modifying effect of chronological age showed that vascular aging categories in middle-aged participants have stronger predictive ability for the risk of cardiovascular events than that in older participants. MACE, a composite of non-fatal myocardial infarction, non-fatal stroke, and cardiovascular mortality; normal VA, normal vascular aging; EVA, early vascular aging; SUPERNOVA, supernormal vascular aging

The modifying effect of chronological age showed that vascular aging categories in middle-aged participants have stronger predictive ability for the risk of cardiovascular events than that in older participants. MACE, a composite of non-fatal myocardial infarction, non-fatal stroke, and cardiovascular mortality; normal VA, normal vascular aging; EVA, early vascular aging; SUPERNOVA, supernormal vascular aging

## Abstract (web summary)

The modifying effect of chronological age showed that vascular aging categories in middle-aged participants have stronger predictive ability for the risk of cardiovascular events than that in older participants. MACE, a composite of non-fatal myocardial infarction, non-fatal stroke, and cardiovascular mortality; normal VA, normal vascular aging; EVA, early vascular aging; SUPERNOVA, supernormal vascular aging.

## Introduction

Vascular aging represents a phenotype characterized by functional and structural damage to the arterial wall, which is a major contributor to cardiovascular diseases (CVD) [1, 2]. As an indicator of vascular aging, vascular age (VA) has been proven to improve cardiovascular risk prediction as an alternative to chronological age [3–6]. Based on the difference between VA and chronological age, previous studies further proposed the concepts of early vascular aging (EVA), normal vascular aging (normal VA), and supernormal vascular aging (SUPERNOVA), which are in favor of identifying potential high- and low- cardiovascular risk subgroups [7–10].

The above studies included older and middle-aged participants to estimate the cardiovascular risk. Whether chronological age affects the ability of vascular aging model to predict the risk of cardiovascular events is unknown. In fact, cardiovascular risk prediction such as SCORE is known to be driven primarily by chronological age, which means that young participants with a series of cardiovascular risk factors may still have a low cardiovascular risk score, that is not precisely accurate for the assessment of vascular status [11]. Several studies showed a higher prevalence of EVA in young participants than that in older people [12, 13], which suggests the applicability discrepancy of vascular aging categories in different chronological age subgroups. Besides, some scholars have conducted the researches of vascular aging model only in specific chronological age groups, such as in middle-aged population or older population respectively [9, 14, 15]. Therefore, the modifying effect of chronological age in total participants needs to be determined.

The study aims to compare the predictability of residuals between VA and chronological age for cardiovascular risk in different chronological age subgroups, examine the modifying effect of chronological age in a community-based population in Beijing, China, and finally determine the applicable population of VA model.

Point of view

- Clinical relevance
- Vascular age assessment has better applicability in middle-aged population than older population.
- Future direction
- Whether the vascular aging indicators can be incorporated into cardiovascular risk prediction models to optimize them requires further investigation.
- Consideration for the Asian population
- The applicability of vascular age assessment should be validated in other Asian countries.

## Methods

### Study population

The participants were recruited from an atherosclerosis cohort conducted in the Pingguoyuan and Gucheng communities in Beijing, China. The cohort initially investigated residents ≥40 years old from December 2011 to April 2012 and followed up until now, which has been reported in detail previously [16]. In brief, a total of 9540 participants were reviewed onsite. Among them, 889 participants without complete baseline information and 488 participants with pre-existing acute myocardial infarction or stroke were excluded. Finally, a total of 8163 participants were included in the analysis. According to World Health Organization’s criteria and definition of the older people in China, we divided the population into <60 years group and ≥60 years group [17, 18].

The Human Research Ethics Committee of Peking University First Hospital approved this study. The protocols were conducted in accordance with institutional guidelines and followed the principles of the Declaration of Helsinki. Written informed consent was obtained from all participants.

### Data collection

The methods of baseline data collection of this cohort study have been described elsewhere [19]. In detail, uniformly trained researchers adopted standardized questionnaires for interviewing all participants’ information about sociodemographic status, education, occupation, diet, lifestyle, health behaviors, and medical history.

Current smoking was defined as smoking at least one cigarette per day for at least half a year. Body mass index (BMI) was calculated by dividing body weight (in kg) by the square of height (in m). Peripheral blood pressure was measured by well-trained researchers using an Omron HEM-7117 electronic sphygmomanometer following a standard method, and the average value of three measurements was used for analysis.

Blood samples were taken from participants in a fasting state for at least 12 h in the morning. The Roche C8000 Automatic Biochemical Analyzer was used to measure indicators, including fasting blood glucose (FBG), total cholesterol (TC), low-density lipoprotein cholesterol (LDL-C), triglycerides (TG), high-density lipoprotein cholesterol (HDL-C), serum creatinine. Estimated glomerular filtration rate (eGFR) was calculated by the Chronic Kidney Disease Epidemiology Collaboration equation [20]. All operations were performed in accordance with standardized operating procedures and were carried out by trained and experienced operators.

Hypertension was defined as a systolic blood pressure (SBP) of ≥140 mmHg or a diastolic blood pressure (DBP) of ≥90 mmHg or any self-reported history of hypertension or current treatment with anti-hypertensive medications. Diabetes mellitus was defined as an FBG concentration of ≥7.0 mmol/L, a 2-h oral glucose tolerance test concentration of ≥11.1 mmol/L, or any self-reported history of diabetes mellitus or current treatment with anti-diabetes medications. Dyslipidemia was defined as a TG concentration of ≥1.7 mmol/L (150 mg/dL), a TC concentration of ≥5.18 mmol/L (200 mg/dL), an LDL-C concentration of ≥3.37 mmol/L (130 mg/dL), an HDL-C concentration of <1.04 mmol/L (40 mg/dL), or any self-reported history of hyperlipidemia, or current treatment with lipid-lowering medications. CVD was defined as a self-reported history of myocardial infarction or stroke (including transient ischemic attack). A family history of CVD was defined as at least a first-degree relative (including parents, siblings, and children) with CVD.

The 10-year CVD risk was estimated using the China-PAR model. Variables in the China-PAR model include sex, chronological age, geographic region (northern or southern China, as divided by the Yangtze River), urbanization (urban or rural), waist circumference, TC, HDL-C, treated or untreated SBP, diabetes, current smoker, and family history of CVD [21].

### Definition of cardiovascular events

The major adverse cardiovascular event (MACE) was a composite of non-fatal myocardial infarction, non-fatal stroke, and cardiovascular mortality. Data on all participants’ myocardial infarction, stroke, and cardiovascular mortalitys until Dec 31, 2021 were collected from the Chinese Center for Disease Control and Prevention (National Mortality Surveillance System) and the Beijing Municipal Health Commission (Inpatient Medical Record Home Page System). The International Classification of Diseases in 10th Revision was used to classify the leading cause of mortality, myocardial infarction and stroke (Supplementary Table 1).

### Measurement of brachial-ankle pulse wave velocity

Brachial-ankle pulse wave velocity (baPWV) was measured using an automatic waveform analyzer (BP-203RPE III, Colin-Omron, Co., Ltd., Tokyo, Japan) by trained operators following standardized protocols. All participants have more than 5 min of rest in an examination room with quiet and temperature control. Then, they were asked to be in the supine position, and four cuffs were wrapped around the bilateral brachia and ankles, which connected to a plethysmographic sensor and oscillometric pressure sensor. The pulse waveforms were then recorded, and the baPWV value was automatically generated. The higher baPWV of both the left and right sides was used for subsequent analyses after examination.

### Vascular age calculation and definition of EVA and SUPERNOVA

VA was defined as the predicted age in a multivariable regression model that included classical cardiovascular risk factors, treatment and baPWV. A backward stepwise approach made the variable selection with multicollinearity checked by variable inflation factors (VIF, variables excluded when VIF > 10). Variables showing a nonlinear relationship with chronological age were transformed by smoothing splines using generalized additive models (knots chosen by 10-fold cross-validation). The above variable selection process was performed in the total population, <60 years group, and ≥60 years group respectively.

The final variables for calculating VA in total population include sex, SBP, DBP, FBG, TG, HDL-C, LDL-C, waist circumference, BMI, current smoking, heart rate, hypertension, diabetes and dyslipidemia, treatment of hypertension, treatment of dyslipidemia, and baPWV. The final variables for calculating VA in <60 years group include sex, SBP, DBP, HDL-C, LDL-C, waist circumference, BMI, current smoking, heart rate, dyslipidemia, treatment of hypertension, treatment of dyslipidemia, and baPWV. And the final variables for calculating VA in ≥60 years group include sex, SBP, DBP, FBG, TG, waist circumference, BMI, current smoking, hypertension, and baPWV.

Then, ∆-age was estimated as the residuals by regressing VA on chronological age [22–24]. The 10th and 90th percentiles of ∆-age were used as cutoffs to define SUPERNOVA, normal VA, and EVA groups, respectively.

### Statistical analyses

The baseline characteristics of participants were expressed as median with interquartile range and compared between groups using the Kruskal-Wallis rank test for continuous variables as appropriate. For categorical variables, data were expressed as frequency (percentage) and compared between groups using the chi-square test as appropriate.

The association between ∆-age as a continuous variable or vascular aging categories as three groups (SUPERNOVA, normal VA, and EVA) and outcomes were assessed by Cox proportional hazards regression model in total participants, <60 years participants, and ≥60 years participants, respectively. Kaplan-Meier curves were performed to observe the association between vascular aging categories and MACE. Covariates in Model 1 included chronological age and sex; those in Model 2 included traditional cardiovascular risk factors (including chronological age, sex, current smoking, BMI, hypertension, diabetes, dyslipidemia, anti-hypertensive treatment, lipid-lowering treatment, hypoglycemic treatment, family history of CVD, and eGFR). And in Model 3, covariates included chronological age, sex and China-PAR score. Interaction tests were performed to determine the influence of chronological age for the predictive effect of ∆-age on the risk of cardiovascular events.

A two-tailed p-value of <0.05 was considered statistically significant in all analyses. R software (version 4.3.2, http://www.R-project.org/) was used to perform the statistical analyses.

## Results

### Study participants and baseline characteristics

A total of 8163 participants finally participated in the study during the median 9.9-year follow-up period. There were 818 (10.0%) composite endpoints observed, of which 157 (1.9%) were acute myocardial infarction, 130 (1.6%) cardiovascular mortality, and 644 (7.9%) stroke. The median of chronological age was 55.0 (50.5–61.0). 2888 (35.4%) participants were male. There were 5691 participants in <60 years group and 2472 in ≥60 years group. The medians of VA in total population, <60 years group, and ≥60 years group were 55.6 (52.2–60.4) years, 51.9 (50.5–53.4) years, and 67.3 (65.0–69.7) years, respectively. According to the 10th and 90th percentile of ∆-age, vascular aging categories in total population were defined as SUPERNOVA (∆-age < −5.40 years), normal VA (−5.40 years ≤ ∆-age ≤ 5.87 years), and EVA (∆-age > 5.87 years); vascular aging categories in <60 years group were defined as SUPERNOVA (∆-age < −2.57 years), normal VA (−2.57 years ≤ ∆-age ≤ 2.48 years), and EVA (∆-age > 2.48 years); vascular aging categories in ≥60 years group were defined as SUPERNOVA (∆-age < −3.41 years), normal VA (−3.41 years ≤ ∆-age ≤ 3.53 years), and EVA (∆-age > 3.53 years).

The clinical characteristics of the participants in the ≥60 years group and <60 years groups are shown in Table 1. There were larger waist circumferences, higher proportions of males, hypertension, diabetes, dyslipidemia, higher CVD risk and baPWV value in the ≥60 years group. Higher percentage of current smoking and higher eGFR level were found in the <60 years group. And the baseline characteristics of three vascular aging categories in the <60 years participants and ≥60 years participants were showed in Supplementary Table 2 and Supplementary Table 3.

**Table 1:** Baseline clinical characteristics of chronological age groups

Variables	Overall Population (*n* = 8163)	<60 years (*n* = 5691)	≥60 years (*n* = 2472)	*P* Value
Chronological age, year	55.0 (50.5–61.0)	53.0 (49.0–56.0)	66.0 (62.0–72.0)	<0.001
Vascular age, year	55.6 (52.2–60.4)	53.8 (51.1–57.0)	61.8 (57.7–66.3)	<0.001
∆-age, year	−0.26 (−3.0 to 2.8)	−0.36 (−2.9 to 2.5)	0.10 (−3.3 to 3.8)	0.005
Male, n (%)	2888 (35.4)	1861 (32.7)	1027 (41.5)	<0.001
BMI, kg/m^2^	25.8 (23.6–28.1)	25.8 (23.6–28.0)	25.9 (23.6–28.2)	0.308
WC, cm	82 (77–88)	82 (76–87)	84 (79–90)	<0.001
Current smoking, n (%)	1551 (19.0)	1197 (21.0)	354 (14.3)	<0.001
SBP, mmHg	131.3 (121.3–143.0)	128.7 (119.7–139.7)	137.7 (127.7–149.7)	<0.001
DBP, mmHg	74.3 (68.0–81.0)	75.3 (69.0–82.0)	72.0 (65.3–79.0)	<0.001
Heart rate, bpm	77.7 (70.7–85.0)	77.3 (70.7–84.7)	78.0 (70.6–85.7)	0.11
TC, mmol/L	5.3 (4.7–5.9)	5.3 (4.7–5.9)	5.3 (4.6–6.0)	0.929
TG, mmol/L	1.3 (0.9–1.9)	1.3 (0.9–1.9)	1.3 (1.0–1.8)	0.378
HDL-C, mmol/L	1.4 (1.2–1.7)	1.4 (1.2–1.7)	1.4 (1.2–1.7)	0.839
LDL-C, mmol/L	3.2 (2.7–3.8)	3.2 (2.7–3.8)	3.3 (2.7–3.8)	0.186
FBG, mmol/L	5.6 (5.2–6.3)	5.6 (5.2–6.2)	5.8 (5.4–6.7)	<0.001
eGFR, mL/min/1.73 m^2^	97.4 (88.1–103.7)	101.0 (95.0–105.8)	87.6 (77.7–93.8)	<0.001
Hypertension, n (%)	3872 (47.4)	2236 (39.3)	1636 (66.2)	<0.001
Diabetes, n (%)	1930 (23.6)	1091 (19.2)	839 (33.9)	<0.001
Dyslipidemia, n (%)	5790 (70.9)	3965 (69.7)	1825 (73.8)	<0.001
Anti-hypertensive medications, n (%)	2415 (29.6)	1312 (23.1)	1103 (44.6)	<0.001
Anti-diabetes medications, n (%)	794 (9.7)	416 (7.3)	378 (15.3)	<0.001
Lipid-lowering medications, n (%)	725 (8.9)	398 (7.0)	327 (13.2)	<0.001
CVD risk, %	5.2 (2.6–10.3)	3.6 (2.0–6.7)	11.4 (7.2–17.2)	<0.001
baPWV, m/s	15.7 (13.9–18.2)	14.9 (13.4–16.8)	18.4 (16.1–21.2)	<0.001

Data were presented as median (IQR) or n (%) of the group. ∆-age indicates the residuals by regressing vascular age on chronological age

*BMI* body mass index, *WC* waist circumference, *SBP* systolic blood pressure, *DBP* diastolic blood pressure, *TC* total cholesterol, *TG* triglycerides, *HDL-C* high-density lipoprotein cholesterol, *LDL-C* low-density lipoprotein cholesterol, *FBG* fasting blood glucose, *eGFR* estimated glomerular filtration rate, *CVD risk* risk of cardiovascular disease estimated by China-PAR model, *baPWV* brachial-ankle pulse wave velocity

### The association between ∆-age and the risk of cardiovascular events in total participants

∆-age as a continuous variable in all participants was significantly and increasingly associated with the risk of MACE after adjusting for traditional cardiovascular risk factors, and every 1-year increase was associated with a rise of 3% in MACE risk (hazard ratio [HR]: 1.03, 95% confidence interval [CI]: 1.02-1.05, p < 0.001; Table 2). When ∆-age was categorized into three vascular aging categories, the participants in the EVA group experienced a higher risk of MACE compared with the normal VA group (HR: 1.36, 95%CI: 1.13–1.65, p < 0.001). In secondary endpoint analysis, ∆-age as a continuous variable was significantly associated with the risk of stroke (HR: 1.03, 95%CI: 1.01–1.05, p = 0.002) and acute myocardial infarction (HR: 1.04, 95%CI: 1.01–1.08, p = 0.025). When ∆-age was categorized into three vascular aging categories, the EVA group showed a 34% higher risk of stroke (HR: 1.34, 95%CI: 1.08–1.65, p = 0.007). Although no significant association between ∆-age and the risk of cardiovascular mortality was found (HR: 1.02, 95%CI: 0.98–1.06, p = 0.235), there was a tendency to increase the risk of cardiovascular mortality in EVA group and decrease the risk of cardiovascular mortality in SUPERNOVA group (EVA group: HR: 1.32, 95%CI: 0.84-2.07; SUPERNOVA group: HR: 0.78, 95%CI: 0.40–1.53) (Table 2).

**Table 2:** Cox proportional hazards regression analyses for the association between vascular aging indicators and cardiovascular events in total participants

Outcomes	Vascular Aging Categories	Event n (%)	Model	
			HR (95%CI)	*P* value
MACE	∆-age, per 1 year	818 (10)	1.03 (1.02–1.05)	<0.001
	SUPERNOVA	66 (8.1)	0.86 (0.66–1.12)	0.255
	Normal VA	608 (9.3)	reference	-
	EVA	144 (17.6)	1.36 (1.13–1.65)	0.001
Stroke	∆-age, per 1 year	644 (7.9)	1.03 (1.01–1.05)	0.002
	SUPERNOVA	50 (6.1)	0.86 (0.64–1.17)	0.340
	Normal VA	479 (7.3)	reference	-
	EVA	115 (14.1)	1.34 (1.08–1.65)	0.007
AMI	∆-age, per 1 year	157 (1.9)	1.04 (1.01–1.08)	0.025
	SUPERNOVA	14 (1.7)	0.88 (0.49–1.55)	0.649
	Normal VA	116 (1.8)	reference	-
	EVA	27 (3.3)	1.41 (0.91–2.19)	0.123
CVD mortality	∆-age, per 1 year	130 (1.6)	1.02 (0.98–1.06)	0.235
	SUPERNOVA	10 (1.2)	0.78 (0.40–1.53)	0.470
	Normal VA	94 (1.4)	reference	-
	EVA	26 (3.2)	1.32 (0.84–2.07)	0.234

Model was adjusted for chronological age, sex, current smoking, body mass index, hypertension, diabetes, dyslipidemia, anti-hypertensive treatment, lipid-lowering treatment, hypoglycemic treatment, family history of cardiovascular disease, and estimated glomerular filtration rate

*HR* hazard ratio, *MACE* a composite of non-fatal acute myocardial infarction, non-fatal stroke, and cardiovascular mortality, *CVD* cardiovascular disease, *AMI* acute myocardial infarction, ∆-age indicates the residuals by regressing vascular age on chronological age, *SUPERNOVA* supernormal vascular aging, *normal VA* normal vascular aging, *EVA* early vascular aging

### The association between ∆-age and cardiovascular events in different chronological age subgroups

**Fig. 1:**
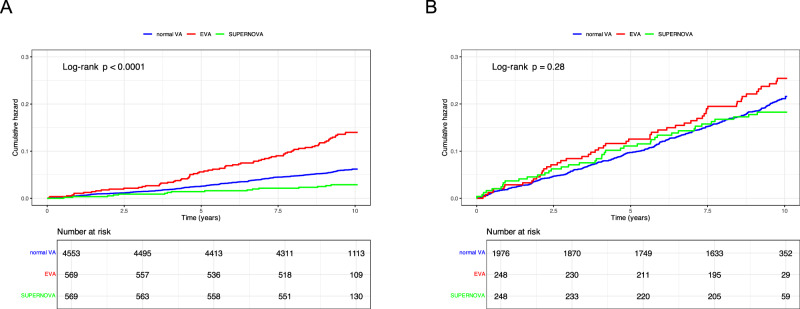
Cumulative hazard of MACE Stratified by Vascular Aging Categories in <60 years group and ≥60 years group. The significant difference of association between vascular aging categories and MACE was found in <60 years group (**A**) and not found in ≥60 years group (**B**) MACE, a composite of non-fatal myocardial infarction, non-fatal stroke, and cardiovascular mortality; normal VA normal vascular aging, EVA early vascular aging, SUPERNOVA supernormal vascular aging

The Kaplan-Meier curve showed participants in the EVA group experienced a higher risk of MACE than participants in the normal VA group, and the SUPERNOVA group experienced a lower risk of MACE during the follow-up period in the <60 years group (P < 0.0001). A significant difference in the association between vascular aging categories and MACE was not found in the ≥60 years group (Fig. 1). Similar significant differences were observed in secondary endpoints of stroke/acute myocardial infarction but not CVD mortality (Supplementary Fig. 1–3.)

In the Cox proportional hazard regression model for the <60 years group, ∆-age as a continuous variable was significantly and increasingly associated with the risk of MACE after adjusting for chronological age and sex, and every 1-year increase was associated with a rise of 22% in MACE risk (HR: 1.22, 95% CI: 1.16–1.29, p < 0.001; Table 3). After adjusting for traditional cardiovascular risk factors, ∆-age was still significantly associated with the risk of MACE (HR: 1.13, 95% CI: 1.07–1.21, p < 0.001; Table 3). Besides, Model 3 showed that ∆-age has an additional risk of cardiovascular events based on chronological age, sex and CVD score (HR: 1.16, 95% CI: 1.09–1.22, p < 0.001; Table 3).

**Table 3:** Cox proportional hazards regression analyses for the association between vascular aging indicators and cardiovascular events in chronological age subgroups

Outcomes	Event n (%)	Model 1		P value for interaction	Model 2		P value for interaction	Model 3		P value for interaction
		HR (95%CI)	P value		HR (95%CI)	P value		HR (95%CI)	P value	
**MACE**				0.010			0.017			0.002
**<60 years group**										
∆-age, per 1 year	358(6.3)	1.22 (1.16–1.29)	<0.001		1.13 (1.07–1.21)	<0.001		1.16 (1.09–1.22)	<0.001	
SUPERNOVA	16 (2.8)	0.52 (0.31–0.85)	0.010		0.61 (0.37–1.02)	0.061		0.56 (0.34–0.93)	0.026	
Normal VA	268 (5.9)	reference	-		reference	-		reference	-	
EVA	74 (13)	2.27 (1.75–2.94)	<0.001		1.63 (1.22–2.16)	0.001		1.81 (1.39–2.36)	<0.001	
**≥60 years group**										
∆-age, per 1 year	460 (18.6)	1.03 (1.00–1.07)	0.046		1.03 (0.99–1.06)	0.148		1.02 (0.99–1.06)	0.204	
SUPERNOVA	41 (16.5)	0.93 (0.67–1.28)	0.645		0.93 (0.66–1.29)	0.645		0.96 (0.69–1.33)	0.805	
Normal VA	365 (18.5)	reference	-		reference	-		reference	-	
EVA	54 (21.8)	1.18 (0.89–1.57)	0.26		1.17 (0.88–1.57)	0.282		1.12 (0.84–1.49)	0.439	
**Stroke**				0.016			0.031			0.004
**<60 years group**										
∆-age, per 1 year	288 (5.1)	1.23 (1.15–1.30)	<0.001		1.13 (1.05–1.21)	0.001		1.17 (1.10–1.24)	<0.001	
SUPERNOVA	12 (2.1)	0.47 (0.26–0.84)	0.011		0.57 (0.31–1.02)	0.060		0.51 (0.28–0.91)	0.022	
Normal VA	217 (4.8)	reference	-		reference	-		reference	-	
EVA	59 (10.4)	2.22 (1.67–2.96)	<0.001		1.50 (1.09–2.07)	0.012		1.80 (1.34–2.43)	<0.001	
**≥60 years group**										
∆-age, per 1 year	356 (14.4)	1.03 (0.99–1.07)	0.160		1.01 (0.97–1.05)	0.586		1.02 (0.98–1.06)	0.448	
SUPERNOVA	36 (14.5)	1.07 (0.75–1.51)	0.723		1.13 (0.79–1.61)	0.505		1.10 (0.78–1.56)	0.586	
Normal VA	278 (14.1)	reference	-		reference	-		reference	-	
EVA	42 (16.9)	1.22 (0.88–1.68)	0.234		1.17 (0.84–1.63)	0.348		1.15 (0.83–1.60)	0.388	
**AMI**				0.201			0.223			0.132
**<60 years group**										
∆-age, per 1 year	67 (1.2)	1.22 (1.08–1.38)	0.001		1.19 (1.03–1.37)	0.022		1.14 (1.01–1.30)	0.036	
SUPERNOVA	4 (0.7)	0.77 (0.28–2.15)	0.621		0.89 (0.32–2.51)	0.826		0.86 (0.31–2.39)	0.773	
Normal VA	47 (1)	reference	-		reference	-		reference	-	
EVA	16 (2.8)	2.72 (1.54–4.79)	0.001		2.46 (1.30–4.63)	0.005		2.07 (1.15–3.74)	0.015	
**≥60 years group**										
∆-age, per 1 year	90 (3.6)	1.06 (0.98–1.14)	0.139		1.07 (0.99–1.15)	0.103		1.04 (0.97–1.13)	0.278	
SUPERNOVA	6 (2.4)	0.70 (0.31–1.62)	0.411		0.63 (0.27–1.46)	0.281		0.74 (0.32–1.70)	0.471	
Normal VA	72 (3.6)	reference	-		reference	-		reference	-	
EVA	12 (4.8)	1.27 (0.69–2.34)	0.449		1.35 (0.72–2.53)	0.346		1.19 (0.64–2.19)	0.584	
**CVD mortality**				0.300			0.291			0.242
**<60 years group**										
∆-age, per 1 year	27 (0.5)	1.23 (1.01–1.50)	0.036		1.17 (0.93–1.46)	0.180		1.13 (0.93–1.37)	0.220	
SUPERNOVA	1 (0.2)	0.46 (0.06–3.42)	0.446		0.44 (0.06–3.37)	0.428		0.51 (0.07–3.84)	0.516	
Normal VA	20 (0.4)	reference	-		reference	-		reference	-	
EVA	6 (1.1)	2.34 (0.94–5.82)	0.069		1.77 (0.64–4.91)	0.274		1.66 (0.65–4.26)	0.291	
**≥60 years group**										
∆-age, per 1 year	103 (4.2)	1.03 (0.96–1.11)	0.385		1.02 (0.94–1.09)	0.664		1.02 (0.95-1.09)	0.645	
SUPERNOVA	9 (3.6)	0.89 (0.45–1.76)	0.731		0.93 (0.46–1.88)	0.835		0.92 (0.46–1.83)	0.809	
Normal VA	83 (4.2)	reference	-		reference	-		reference	-	
EVA	11 (4.4)	1.01 (0.54–1.89)	0.981		0.94 (0.50–1.78)	0.852		0.95 (0.51–1.78)	0.874	

Model 1 was adjusted for chronological age and sex; model 2 was adjusted for chronological age, sex, current smoking, body mass index, hypertension, diabetes, dyslipidemia, anti-hypertensive treatment, lipid-lowering treatment, hypoglycemic treatment, family history of cardiovascular disease, and estimated glomerular filtration rate; model 3 was adjusted for chronological age, sex and CVD score from China-PAR

*HR* hazard ratio, *MACE* a composite of non-fatal acute myocardial infarction, non-fatal stroke, and cardiovascular mortality, *CVD* cardiovascular disease, *AMI* acute myocardial infarction, *∆-age* indicates the residuals by regressing vascular age on chronological age, *SUPERNOVA* supernormal vascular aging, *normal VA* normal vascular aging, *EVA* early vascular aging

In the Cox proportional hazard regression model for the ≥60 years group, ∆-age as a continuous variable was increasingly associated with the risk of MACE after adjusting for chronological age and sex (HR: 1.03, 95% CI: 1.00–1.07, p = 0.046). However, the association was not significant when adjusting for traditional cardiovascular risk factors (HR: 1.03, 95% CI: 0.99-1.06, p = 0.148). And the additional risk of cardiovascular events based on chronological age, sex and CVD score was not found in Model 3 (HR: 1.02, 95% CI: 0.99–1.06, p = 0.204; Table 3).

When ∆-age was categorized into three vascular aging categories for the <60 years group, the subjects in the EVA group experienced a higher risk of MACE compared with normal VA groups after adjusting for chronological age and sex (HR: 2.27, 95%CI: 1.75–2.94, p < 0.001), and the SUPERNOVA group was associated with a 48% risk reduction of MACE (HR: 0.52, 95% CI: 0.31–0.85, p = 0.010). After adjusting for traditional cardiovascular risk factors, the EVA group still had a statistically increased risk of MACE (HR: 1.63, 95% CI: 1.22–2.16, p = 0.001) compared with the normal VA group. Even after adjusting for chronological age, sex, and CVD score in Model 3, the EVA group was associated with an additional risk of MACE (HR: 1.81, 95%CI: 1.39–2.36, p < 0.001), and the SUPERNOVA group showed a further risk reduction of MACE (HR: 0.56, 95%CI: 0.34–0.93, p = 0.026). However, the similar results were not shown in ≥60 years group (Table 3).

In secondary endpoint analysis, ∆-age as a continuous variable was increasingly associated with the risk of stroke (HR: 1.23, 95% CI: 1.15–1.30, p < 0.001), acute myocardial infarction (HR: 1.22, 95% CI: 1.08-1.38, p = 0.001) and cardiovascular mortality (HR: 1.23, 95% CI: 1.01–1.50, p = 0.036) after adjusting for chronological age and sex in <60 years group (Table 3). When Δ-age was categorized into three vascular aging categories, the EVA groups still showed higher risks of stroke and acute myocardial infarction in both Model 2 and Model 3 but not of CVD mortality. However, no significant association differences between vascular aging categories and stroke/acute myocardial infarction/CVD mortality were found in the ≥60 years group (Table 3).

Interaction analysis showed that chronological age significantly modified the relationship between ∆-age and the risk of MACE and of stroke (Table 3).

## Discussion

This prospective cohort study showed that ∆-age was associated with cardiovascular events in total participants, and chronological age significantly modified the relationship between ∆-age and the risk of MACE. After chronological age stratification, ∆-age and vascular aging categories in middle-aged participants have stronger predictive ability for the risk of MACE, stroke and acute myocardial infarction than that in older participants. And ∆-age has an additional risk of cardiovascular events based on CVD score in middle-aged participants. This study suggests that VA assessment may have better applicability in middle-aged participants.

Vascular aging increasing the thickness of intimal and medial layers, results in vascular function and structure injury, and finally leads to arterial stiffness [25]. A variety of factors, such as chronological age, blood pressure, inflammation, adiposity, impaired glucose homeostasis, metabolic syndrome, and unhealthy lifestyle, contribute to arterial stiffness [15, 26, 27]. VA, a concept for reflecting structural and functional changes of blood vessels, is an indicator of arterial stiffness and a beneficial tool to improve adherence since it directly reflects the cardiovascular risk related to the status of vascular trees [25, 28]. Based on residuals by regressing VA on chronological age, ∆-age indicates the degree of vascular aging on the basis of current chronological age [24]. As shown in this study, ∆-age were increasingly associated with the risk of MACE after adjusting for traditional cardiovascular risk factors (Model 2) or CVD score (Model 3) in total participants, which is consistent with other researches [7–9].

Chronological age is not an adequate marker for an individual to evaluate the functional status and the risk of CVD. There is heterogeneity among the older population for that the aging process includes healthy vascular aging, or aging with various chronic diseases [15, 29, 30], such as hypertension, diabetes, dyslipidemia, kidey disease and so on. The latter has a more complicated situation and is associated with a higher risk of cardiovascular events. As shown in our baseline characteristics, a higher proportion of cardiovascular risk factors was found in the ≥60 years group compared with <60 years group. Therefore, compared with middle-aged population, more comprehensive cardiovascular risk conditions need to be considered when making the cardiovascular risk prediction for older people, which means we cannot rely on a certain index or a certain model to evaluate cardiovascular risk in older participants. And unmeasured bias including survivorship bias may lead to the current result in the older people. This performance might explain the results that ∆-age have a poor predictive ability for the risk of MACE in older people.

Previous studies found a higher prevalence of EVA in younger participants [12, 13], where the EVA definition was based on PWV. PWV is recognized as an index of arterial stiffness and is the most commonly used method for the assessment of arterial stiffness [31]. Studies have shown changes in PWV in different chronological age groups and confirmed a gradual increase of PWV with chronological age [13, 32], which is in line with our result showing higher baPWV in the ≥60 years group. However, there is a nonlinear progression of PWV with chronological age, and the power of PWV to predict CVD and mortality events was stronger in younger and middle-aged participants than that in older ones [33]. In the present study, VA was defined by classical cardiovascular risk factors and baPWV, which may also explains why ∆-age based on VA have a better predictive ability for the risk of cardiovascular events in middle-aged participants.

Some limitations to our study should be noted. First, we used baPWV instead of carotid-femoral PWV, which is the gold standard for arterial stiffness. However, studies showed that baPWV had a good correlation with carotid-femoral pulse wave velocity, and both were significantly associated with CVD risk factors [34, 35]. Second, the number of older people (*n* = 2472) in our cohort is less than that of middle-aged people (*n* = 5691). Larger studies to elucidate any potential effects of ∆-age in the elderly population may be needed. Third, older people are more likely to have chronic disease such as hypertension and diabetes, making them more susceptible to survivorship bias in research compared to middle-aged adults. Under the current trend of an aging population and the global burden of CVD [36], it is crucial to provide a more comprehensive risk prediction method for older population.

### Perspective of Asia

The high incidence of CVD in Asia makes cardiovascular risk assessment particularly important [37–39]. Vascular aging, a major contributor to CVD, tends to occur at a younger age. Several studies showed a higher prevalence of EVA in young participants than that in older people [12, 13].Therefore, the assessment of vascular aging is of greater value in middle-aged adults for identifying early cardiovascular risk. We suggested the applicable population of VA model in China and provided cohort data support for cardiovascular risk prediction. Whether these results are applicable to other Asian countries needs further verification.

### Conclusion

Our findings indicate that residuals between VA and chronological age were associated with cardiovascular events in total participants. Chronological age significantly modified the relationship between ∆-age and cardiovascular events. After chronological age stratification, ∆-age and vascular aging categories in middle-aged participants have stronger predictive ability for the risk of cardiovascular events than that in older participants. The VA model for the assessment of cardiovascular risk may be more applicable to the middle-aged population.

## Supplementary information

Supplementary Table

Supplementary figure 1

Supplementary figure 2

Supplementary figure 3
