# Supplementary material for: The modifying effect of chronological age on the predictive value of vascular aging indicators for the long-term cardiovascular events risk
Source: Hypertens Res. 2026 Jan 15;49(4):1150–60. doi: 10.1038/s41440-025-02503-6 (PMC13050648; doi:10.1038/s41440-025-02503-6)
Supplement: Supplementary file 1 — Supplementary Table [file 41440_2025_2503_MOESM1_ESM.docx]

**Supplementary Table 1 The source and ICD-10 code of the cardiovascular events in this study**

| Cardiovascular events | Source | ICD-10 code |
| --- | --- | --- |
| Cardiovascular death | The Chinese Center for Disease Control and Prevention-National Mortality Surveillance System | I00-I99 |
| Myocardial infarction | The Beijing Municipal Health Commission-Beijing inpatient medical record home page systerm | Acute ST-segment elevation myocardial infarction (I21.001-006, I21.101-105, I21.201-211, I21.213-230, I21.301, I21.304, I22.001-003, I22.101-103, I22.801-818), Acute non-ST-segment elevation myocardial infarction (I21.401-404), Other myocardial infarction (I21.302, I21.303, I21.305-308, I21.901, I21.902, I21.907, I21.910, I21.911, I22.901) |
|  | The Chinese Center for Disease Control and Prevention-National Mortality Surveillance System | I21.0, I21.1, I21.2, I21.3, I21.4, I21.9, I22.0, I22.1, I22.8, I22.9 |
| Stroke | The Beijing Municipal Health Commission-Beijing inpatient medical record home page system | Ischemic stroke (I63), Hemorrhagic stroke (I60-I61), Other stroke (I64) |
|  | The Chinese Center for Disease Control and Prevention-National Mortality Surveillance System | I60, I61, I63, I64 |

ICD-10: The International Classification of Diseases in 10th Revision.

**Supplementary Table 2 Baseline Clinical Characteristics of vascular aging categories in the <60 years group**

| Variables | Overall population  (n=5691) | SUPERNOVA  (n=569) | Normal VA  (n=4553) | EVA  (n=569) | P value |
| --- | --- | --- | --- | --- | --- |
| Chronological age, y | 53.0(49.0-56.0) | 52.0(48.0-56.0) | 53.0(49.0-56.0) | 53.0(49.0-56.0) | 0.161 |
| Vascular age, y | 51.9(50.5-53.4) | 48.2(47.2-49.3) | 52.0(50.8-53.1) | 55.4(54.6-56.2) | <0.001 |
| ∆-age, y | 0.05(-1.25 to 1.36) | -3.41(-4.23 to -2.96) | 0.05(-0.97 to 1.06) | 3.19(2.81-3.92) | <0.001 |
| Male, n (%) | 1861(32.7) | 135(23.7) | 1511(33.2) | 215(37.8) | <0.001 |
| BMI, kg/m^2^ | 25.8(23.6-28.0) | 25.0(22.4-27.4) | 25.8(23.7-28.1) | 26.2(24.0-28.6) | <0.001 |
| WC, cm | 82.0(76.0-87.0) | 77.0(71.0-84.0) | 82.0(76.2-88.0) | 84.0(80.0-90.0) | <0.001 |
| Current smoking, n (%) | 1197(21.0) | 122(21.4) | 978(21.5) | 97(17.0) | 0.046 |
| SBP, mmHg | 128.7(119.7-139.7) | 118.3(110.0-127.0) | 128.7(120.0-138.7) | 140.7(131.0-152.0) | <0.001 |
| DBP, mmHg | 75.3(69.0-82.0) | 73.0(67.0-80.3) | 75.7(69.7-82.3) | 74.0(68.0-79.7) | <0.001 |
| Heart rate, bpm | 77.3(70.7-84.7) | 79.0(73.0-86.3) | 77.3(71.0-84.7) | 76.0(68.0-83.3) | <0.001 |
| TC, mmol/L | 5.26(4.70-5.92) | 4.78(4.24-5.25) | 5.28(4.73-5.92) | 5.69(5.15-6.30) | <0.001 |
| TG, mmol/L | 1.30(0.90-1.87) | 1.04(0.75-1.47) | 1.31(0.91-1.89) | 1.43(1.04-1.93) | <0.001 |
| HDL-C, mmol/L | 1.40(1.17-1.66) | 1.41(1.16-1.69) | 1.39(1.17-1.65) | 1.42(1.22-1.69) | 0.047 |
| LDL-C, mmol/L | 3.21(2.71-3.76) | 2.73(2.29-3.17) | 3.22(2.74-3.77) | 3.57(3.08-4.06) | <0.001 |
| FBG, mmol/L | 5.55(5.17-6.15) | 5.33(5.05-5.75) | 5.55(5.18-6.13) | 5.87(5.33-6.86) | <0.001 |
| eGFR, ﻿  mL/min/1.73 m^2^ | 101.02(95.03-105.81) | 100.80(94.90-106.06) | 100.94(95.01-105.61) | 101.75(95.39-106.81) | 0.154 |
| Hypertension, n (%) | 2236(39.3) | 93(16.3) | 1703(37.4) | 440(77.3) | <0.001 |
| Diabetes, n (%) | 1091(19.2) | 57(10.0) | 840(18.4) | 194(34.1) | <0.001 |
| Dyslipidemia, n (%) | 3965(69.7) | 229(40.2) | 3217(70.7) | 519(91.2) | <0.001 |
| Anti-hypertensive medications, n (%) | 1312(23.1) | 37(6.5) | 945(20.8) | 330(58.0) | <0.001 |
| Anti-diabetes medications, n (%) | 416(7.3) | 14(2.5) | 300(6.6) | 102(17.9) | <0.001 |
| Lipid-lowering medications, n (%) | 398(7.0) | 11(1.9) | 258(5.7) | 129(22.7) | <0.001 |
| CVD risk, % | 3.60(1.96-6.65) | 2.00(1.06-4.34) | 3.55(2.02-6.48) | 5.81(3.49-10.05) | <0.001 |
| baPWV, m/s | 14.90(13.41-16.75) | 12.19(11.20-13.27) | 14.92(13.61-16.60) | 17.66(15.96-19.88) | <0.001 |

Data were presented as median (IQR) or n (%) of the group. ∆-age indicates the residuals by regressing vascular age on chronological age; BMI, body mass index; WC, waist circumference; SBP, systolic blood pressure; DBP, diastolic blood pressure; TC, total cholesterol; TG, triglycerides; HDL-C, high-density lipoprotein cholesterol; LDL-C, low-density lipoprotein cholesterol; FBG, fasting blood glucose; eGFR, estimated glomerular filtration rate; CVD, cardiovascular disease; baPWV, brachial-ankle pulse wave velocity; SUPERNOVA, supernormal vascular aging; normal VA; normal vascular aging; EVA, early vascular aging.

**Supplementary Table 3 Baseline Clinical Characteristics of vascular aging categories in the ≥60 years group**

| Variables | Overall population  (n=2472) | SUPERNOVA  (n=248) | Normal VA  (n=1976) | EVA  (n=248) | P value |
| --- | --- | --- | --- | --- | --- |
| Chronological age, y | 66.0(62.0-72.0) | 67.0(62.0-72.0) | 66.0(62.0-72.0) | 67.0(63.0-72.0) | 0.645 |
| Vascular age, y | 67.3(65.0-69.7) | 62.7(61.4-64.4) | 67.3(65.4-69.3) | 72.3(70.7-73.9) | <0.001 |
| ∆-age, y | -0.11(-1.94 to 1.88) | -4.33(-5.11 to -3.81) | -0.11(-1.52 to 1.45) | 4.53(4.01-5.64) | <0.001 |
| Male, n (%) | 1027(41.5) | 79(31.9) | 831(42.1) | 117(47.2) | 0.001 |
| BMI, kg/m^2^ | 25.9(23.6-28.2) | 26.4(24.3-28.5) | 25.9(23.7-28.1) | 25.7(23.1-27.9) | 0.012 |
| WC, cm | 84.0(79.0-90.0) | 82.0(78.0-87.0) | 84.0(79.0-90.0) | 84.5(80.0-91.0) | <0.001 |
| Current smoking, n (%) | 354(14.3) | 50(20.2) | 285(14.4) | 19(7.7) | <0.001 |
| SBP, mmHg | 137.7(127.7-149.7) | 131.2(121.9-140.8) | 137.7(127.3-149.0) | 146.3(135.9-158.4) | <0.001 |
| DBP, mmHg | 72.0(65.3-79.0) | 77.8(71.7-84.0) | 72.3(65.7-79.0) | 65.7(60.7-71.1) | <0.001 |
| Heart rate, bpm | 78.0(70.6-85.7) | 78.2(70.0-85.3) | 78.0(70.7-85.7) | 78.7(70.7-89.0) | 0.538 |
| TC, mmol/L | 5.29(4.62-6.01) | 5.54(4.80-6.18) | 5.27(4.62-6.01) | 5.12(4.48-5.74) | <0.001 |
| TG, mmol/L | 1.30(0.97-1.80) | 1.44(1.05-2.07) | 1.29(0.97-1.80) | 1.24(0.91-1.65) | <0.001 |
| HDL-C, mmol/L | 1.39(1.17-1.65) | 1.37(1.16-1.62) | 1.39(1.18-1.66) | 1.40(1.16-1.66) | 0.635 |
| LDL-C, mmol/L | 3.25(2.70-3.82) | 3.42(2.81-4.00) | 3.25(2.71-3.82) | 3.16(2.58-3.67) | 0.001 |
| FBG, mmol/L | 5.80(5.37-6.66) | 5.64(5.27-6.22) | 5.79(5.36-6.66) | 6.06(5.65-6.92) | <0.001 |
| eGFR, ﻿  mL/min/1.73 m^2^ | 87.61(77.66-93.75) | 87.02(76.67-92.62) | 87.53(77.33-93.77) | 89.27(81.07-94.37) | 0.062 |
| Hypertension, n (%) | 1636(66.2) | 115(46.4) | 1304(66.0) | 217(87.5) | <0.001 |
| Diabetes, n (%) | 839(33.9) | 72(29.0) | 657(33.2) | 110(44.4) | 0.001 |
| Dyslipidemia, n (%) | 1825(73.8) | 201(81.0) | 1449(73.3) | 175(70.6) | 0.014 |
| Anti-hypertensive medications, n (%) | 1103(44.6) | 84(33.9) | 877(44.4) | 142(57.3) | <0.001 |
| Anti-diabetes medications, n (%) | 378(15.3) | 26(10.5) | 296(15.0) | 56(22.6) | 0.001 |
| Lipid-lowering medications, n (%) | 327(13.2) | 34(13.7) | 259(13.1) | 34(13.7) | 0.916 |
| CVD risk, % | 11.39(7.22-17.24) | 9.68(5.76-15.88) | 11.28(7.20-16.91) | 14.55(9.50-20.09) | <0.001 |
| baPWV, m/s | 18.41(16.09-21.16) | 15.08(13.63-16.60) | 18.34(16.24-20.81) | 22.67(20.38-26.06) | <0.001 |

Data were presented as median (IQR) or n (%) of the group. ∆-age indicates the residuals by regressing vascular age on chronological age; BMI, body mass index; WC, waist circumference; SBP, systolic blood pressure; DBP, diastolic blood pressure; TC, total cholesterol; TG, triglycerides; HDL-C, high-density lipoprotein cholesterol; LDL-C, low-density lipoprotein cholesterol; FBG, fasting blood glucose; eGFR, estimated glomerular filtration rate; CVD, cardiovascular disease; baPWV, brachial-ankle pulse wave velocity; SUPERNOVA, supernormal vascular aging; normal VA; normal vascular aging; EVA, early vascular aging.

**Supplementary Figure Legend**

**Supplementary Figure 1. Cumulative hazard of Stroke Stratified by Vascular Aging Categories in <60 years group and ≥60 years group**

The significant difference of association between vascular aging categories and stroke was found in <60 years group (A) and not found in ≥60 years group (B)

Abbreviations: normal VA, normal vascular aging; EVA, early vascular aging; SUPERNOVA, supernormal vascular aging.

**Supplementary Figure 2. Cumulative hazard of AMI Stratified by Vascular Aging Categories in <60 years group and ≥60 years group**

The significant difference of association between vascular aging categories and AMI was found in <60 years group (A) and not found in ≥60 years group (B)

Abbreviations: AMI, acute myocardial infarction; normal VA, normal vascular aging; EVA, early vascular aging; SUPERNOVA, supernormal vascular aging.

**Supplementary Figure 3. Cumulative hazard of CVD Mortality Stratified by Vascular Aging Categories in <60 years group and ≥60 years group**

There were no significant differences of association between vascular aging categories and CVD mortality in <60 years group (A) and in ≥60 years group (B)

Abbreviations: CVD, cardiovascular disease; normal VA, normal vascular aging; EVA, early vascular aging; SUPERNOVA, supernormal vascular aging.
