# Supplementary figures and images for: The modifying effect of chronological age on the predictive value of vascular aging indicators for the long-term cardiovascular events risk
Source: Hypertens Res. 2026 Jan 15;49(4):1150–60. doi: 10.1038/s41440-025-02503-6 (PMC13050648; doi:10.1038/s41440-025-02503-6)

A

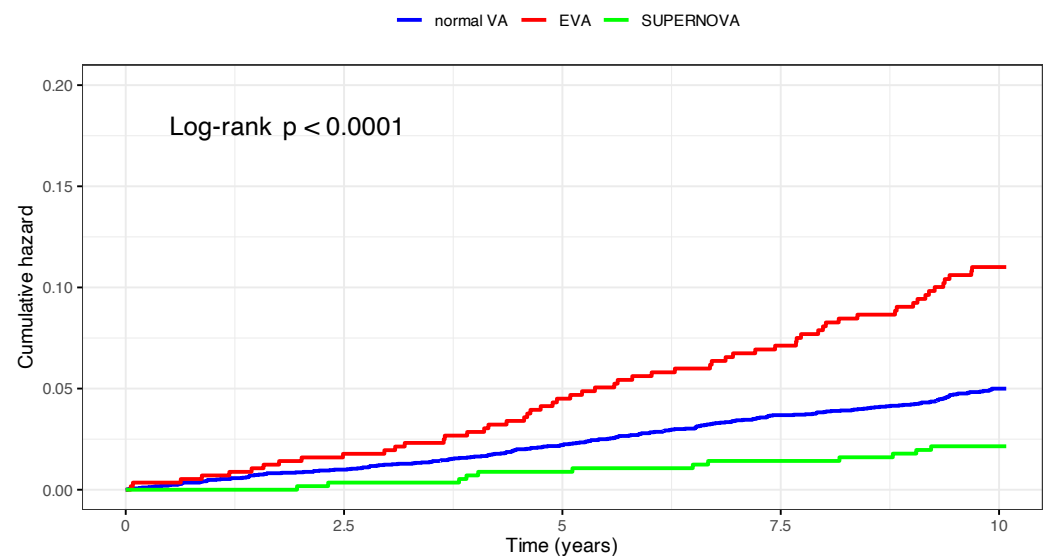

Number at risk

|           |      |      |      |      |      |
|-----------|------|------|------|------|------|
| normal VA | 4553 | 4501 | 4424 | 4338 | 1127 |
| EVA       | 569  | 558  | 541  | 527  | 113  |
| SUPERNOVA | 569  | 566  | 561  | 555  | 131  |

B

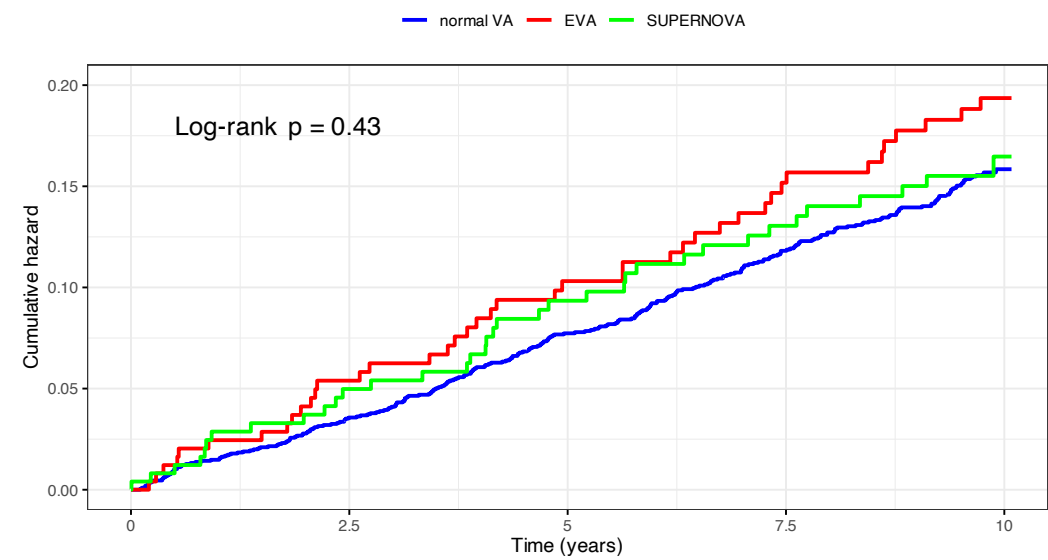

Number at risk

|           |      |      |      |      |     |
|-----------|------|------|------|------|-----|
| normal VA | 1976 | 1885 | 1771 | 1665 | 363 |
| EVA       | 248  | 234  | 215  | 197  | 29  |
| SUPERNOVA | 248  | 235  | 222  | 208  | 60  |

Supplement: Supplementary file 2 — Supplementary figure 1 [file 41440_2025_2503_MOESM2_ESM.pdf]

A

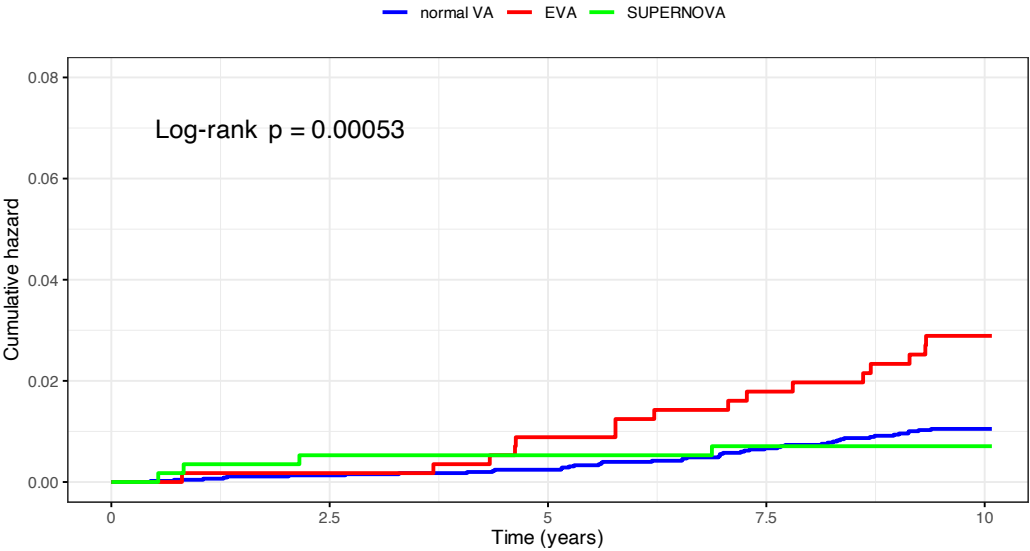

Number at risk

|           |      |      |      |      |      |
|-----------|------|------|------|------|------|
| normal VA | 4553 | 4537 | 4505 | 4456 | 1168 |
| EVA       | 569  | 566  | 558  | 551  | 117  |
| SUPERNOVA | 569  | 565  | 562  | 558  | 132  |

B

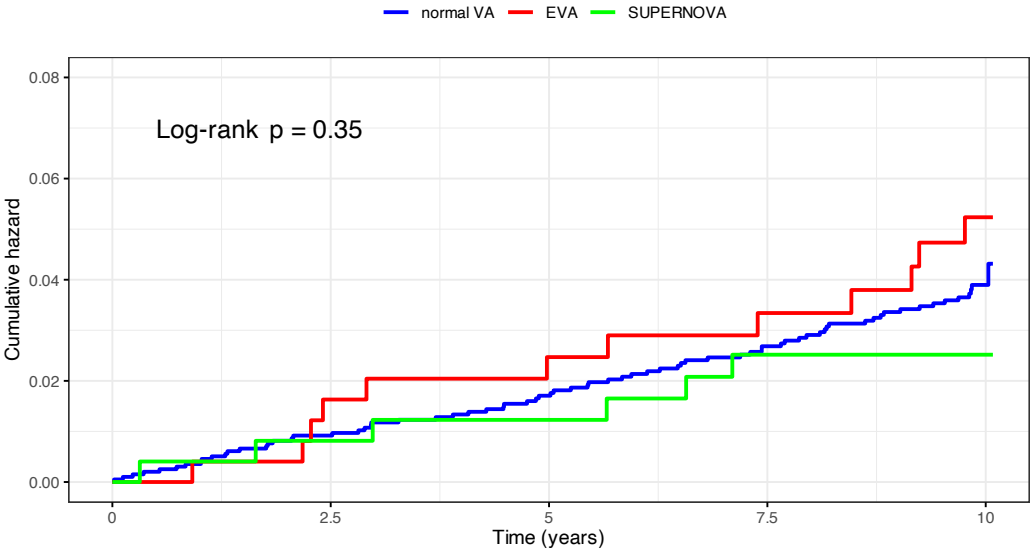

Number at risk

|           |      |      |      |      |     |
|-----------|------|------|------|------|-----|
| normal VA | 1976 | 1933 | 1873 | 1804 | 399 |
| EVA       | 248  | 243  | 234  | 224  | 36  |
| SUPERNOVA | 248  | 242  | 237  | 226  | 64  |

Supplement: Supplementary file 3 — Supplementary figure 2 [file 41440_2025_2503_MOESM3_ESM.pdf]

A

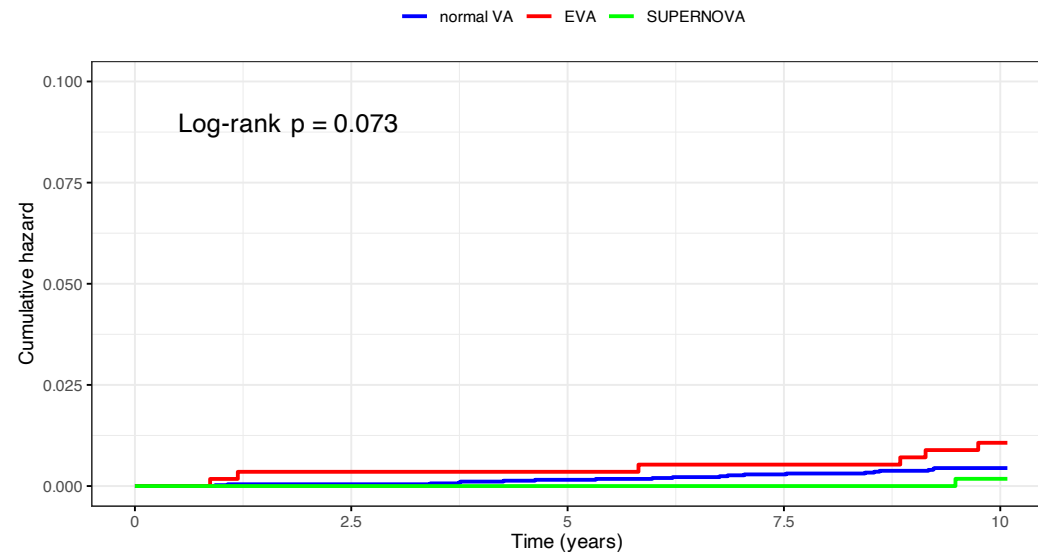

Number at risk

|           |      |      |      |      |      |
|-----------|------|------|------|------|------|
| normal VA | 4553 | 4543 | 4516 | 4483 | 1183 |
| EVA       | 569  | 567  | 563  | 561  | 122  |
| SUPERNOVA | 569  | 568  | 565  | 562  | 133  |

B

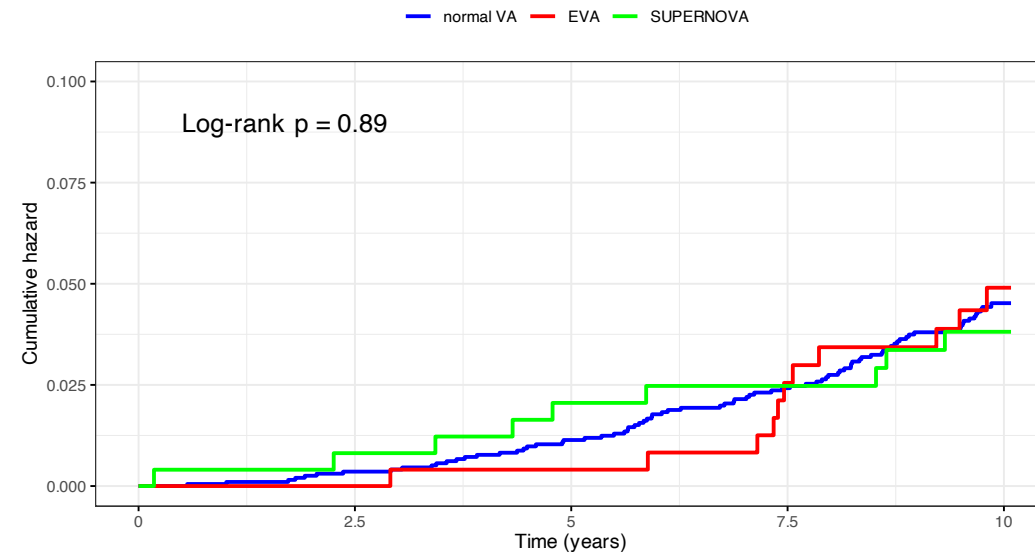

Number at risk

|           |      |      |      |      |     |
|-----------|------|------|------|------|-----|
| normal VA | 1976 | 1948 | 1899 | 1842 | 415 |
| EVA       | 248  | 247  | 239  | 229  | 38  |
| SUPERNOVA | 248  | 244  | 239  | 231  | 66  |

Supplement: Supplementary file 4 — Supplementary figure 3 [file 41440_2025_2503_MOESM4_ESM.pdf]
